# Tumor immune microenvironment of self-identified African American and non-African American triple negative breast cancer

**DOI:** 10.1038/s41523-022-00449-3

**Published:** 2022-07-22

**Authors:** Michal Marczyk, Tao Qing, Tess O’Meara, Vesal Yagahoobi, Vasiliki Pelekanou, Yalai Bai, Emily Reisenbichler, Kimberly S. Cole, Xiaotong Li, Vignesh Gunasekharan, Eiman Ibrahim, Kristina Fanucci, Wei Wei, David L. Rimm, Lajos Pusztai, Kim R. M. Blenman

**Affiliations:** 1 Department of Data Science and Engineering, Silesian University of Technology, Gliwice, Poland; 2 Yale Cancer Center, Yale University, New Haven, CT USA; 3 Department of Internal Medicine, Section of Medical Oncology, Yale University, New Haven, CT USA; 4 Department of Pathology, Yale University, New Haven, CT USA; 5 Department of Computational Biology & Bioinformatics, Biological & Biomedical Sciences, Yale University, New Haven, CT USA; 6 Department of Pharmacology, Yale University, New Haven, CT USA; 7 Department of Biostatistics, Yale University, New Haven, CT USA; 8 Department of Computer Science, Yale University, New Haven, CT USA; 9 Present Address: Department of Internal Medicine, Brigham and Women’s Hospital, Boston, MA USA; 10 Present Address: Precision Medicine – Oncology, Translational Medical Oncology, Translational Medicine Early Development, Sanofi, Cambridge, MA USA; 11 Present Address: Sema4 Genomics, Branford, CT USA

**Keywords:** Breast cancer, Tumour immunology, Transcriptomics, Cancer microenvironment, Translational research

## Abstract

Differences in the tumor immune microenvironment may result in differences in prognosis and response to treatment in cancer patients. We hypothesized that differences in the tumor immune microenvironment may exist between African American (AA) and NonAA patients, due to ancestry-related or socioeconomic factors, that may partially explain differences in clinical outcomes. We analyzed clinically matched triple-negative breast cancer (TNBC) tissues from self-identified AA and NonAA patients and found that stromal TILs, PD-L1 IHC-positivity, mRNA expression of immune-related pathways, and immunotherapy response predictive signatures were significantly higher in AA samples (*p* < 0.05; Fisher’s Exact Test, Mann–Whitney Test, Permutation Test). Cancer biology and metabolism pathways, TAM-M2, and Immune Exclusion were significantly higher in NonAA samples (*p* < 0.05; Permutation Test, Mann–Whitney Test). There were no differences in somatic tumor mutation burden. Overall, there is greater immune infiltration and inflammation in AA TNBC and these differences may impact response to immune checkpoint inhibitors and other therapeutic agents that modulate the immune microenvironment.

## Introduction

An important histologic feature of triple-negative breast cancer (TNBC) is greater immune cell infiltration compared to estrogen receptor-positive cancers. The degree of immune infiltration is the most consistently reported prognostic and chemotherapy treatment response predictive marker in TNBC^1^. Both high stromal tumor-infiltrating lymphocyte (sTILs) scores and high immune-related gene expression, including PD-L1, predict better prognosis and higher pathologic complete response (pCR) rates to neoadjuvant chemotherapy with or without immune checkpoint inhibitors^2–5^. In metastatic TNBC, PD-L1 protein expression is required for benefit from immune checkpoint inhibitors (atezolizumab; pembrolizumab) with chemotherapy^5–8^.

Response rates to neoadjuvant chemotherapy in stage I-III TNBC and survival are unequal between patients of different ancestries. In a large population-wide study, the pCR rate is statistically significantly higher in Non-African American (NonAA) compared to African American (AA) patients with TNBC. This difference persisted even after adjusting for age, clinical and histopathologic factors, comorbidity index, and socioeconomic factors including facility type, geographic region, insurance status, census-derived median income, and length of therapy, suggesting potential biological differences that influence treatment response^9–13^. We hypothesize that differences in the tumor immune microenvironment may exist between AA and NonAA patients, due to ancestry-related or socioeconomic factors, and these differences may partially drive the differences in clinical outcomes. We previously performed gene expression analysis of AA and NonAA patients in The Cancer Genome Atlas (TCGA). We found no consistent differences in AA relative to NonAA TNBC in immune gene expression or CIBERSORT cell types^14^. The purpose of the current study was to further characterize the pretreatment immune microenvironment of patients self-identified as NonAA or AA using multiple analytical methods including germline and somatic whole-exome sequencing (WES), tumor RNA sequencing (RNAseq), and histology analysis (Supplementary Fig. 1).

## Results

### Patient characteristics and analysis sets

The two ancestry cohorts were matched for age, stage, grade, histology, and date of diagnosis, however, AA patients had higher rates of obesity (*p* = 0.045; Fisher’s Exact Test), hypertension (*p* = 0.0046; Fisher’s Exact Test), and type II diabetes mellitus (*p* = 0.0003; Fisher’s Exact Test). Among AA patients 72.2% received adjuvant chemotherapy, among those who received chemotherapy 46.2% received a combination of a taxane and anthracycline. Among the NonAA patients with known treatment history, these proportions were 79.1% and 70.6%, respectively. These differences did not reach statistical significance (*p* = 0.0569; Fisher’s Exact Test) (Table 1). AA patients experienced higher local or distant recurrences (33.3% versus 14.3%; *p* = 0.0238, Fisher’s Exact Test), and a trend for worse recurrence-free survival (*p* = 0.0612; Mantel–Cox Test) (Supplementary Fig. 2).

**Table 1:** Patient characteristics.

Total patients (*n* = 110)	NonAA (*n* = 56)	AA (*n* = 54)	*p*-value^d^
*Ethnicity*			
Hispanic or Latino	0 (0.0%)	1 (1.9%)	0.3657
Not Hispanic or Latino	41 (73.2%)	43 (79.6%)	
Unknown	15 (26.8%)	10 (18.5%)	
*Clinical variables*			
Age (years), median	56.0	59.5	0.795^a^
Interquartile range (years)	49.0–67.3	50.8–66.0	
Follow-up time (years), median	5.9	6.4	0.344^a^
Interquartile range (years)	2.0–10.9	3.0–10.9	
Recurrence rate, n (%)			0.0238^b^
Yes^b^	8 (14.3%)	18 (33.3%)	
No^b^	46 (82.1%)	34 (63.0%)	
Unknown	2 (3.6%)	2 (3.7%)	
Adjuvant chemotherapy, n (%)			0.0569^c^
Anthracycline + Taxane^c^	24 (42.9%)	18 (33.3%)	
Other^c^	10 (17.9%)	21 (38.9%)	
None	9 (16.1%)	15 (27.8%)	
Unknown	12 (21.4%)	0 (0%)	
*Chronic conditions*			
Obesity (BMI > 30), *n* (%)	14 (25.0%)	24 (44.4%)	0.045
Hypertension, *n* (%)	22 (39.3%)	36 (66.7%)	0.0046
Type 2 diabetes mellitus, *n* (%)	5 (8.9%)	21 (38.9%)	0.0003
Autoimmunity (SLE, Sjogren’s Disease, RA, IBD, MS, Type I Diabetes Mellitus, Graves’ or Hashimoto’s Disease), *n* (%)	6 (10.7%)	7 (13.0%)	0.774
Chronic kidney disease, *n* (%)	3 (5.4%)	8 (14.8%)	0.121
Hyperlipidemia, *n* (%)	17 (30.4%)	17 (31.5%)	1.000
*Pathological variables*			
Stage, *n* (%)			0.407
I	22 (39.3%)	20 (37.0%)	
II	28 (50.0%)	32 (59.3%)	
III	6 (10.7%)	2 (3.7%)	
Tumor size			0.269
T1	29	26	
T2	24	24	
T3	1	4	
T4	2	0	
Nodal status			0.488
N0	33	32	
N1	14	18	
N2	4	2	
NA	5	2	
Histological grade, *n* (%)			1.000
Well-differentiated	1 (1.8%)	0 (0.0%)	
Moderately differentiated	10 (17.9%)	9 (16.7%)	
Poorly differentiated	45 (80.4%)	45 (83.3%)	
*Breast cancer biomarkers*			
ESR1 mRNA normalized expression, median (CI)	7.531 (7.000–7.924)	7.548 (7.287–8.435)	0.6339
ERBB2 mRNA normalized expression, median (CI)	11.13 (10.76–11.25)	11.05 (10.90–11.57)	0.8278
PGR mRNA normalized expression, median (CI)	5.661 (5.355–6.133)	6.087 (5.597–6.678)	0.6499
MKI67 mRNA normalized expression, median (CI)	13.57 (12.50–13.62)	13.57 (12.68–13.63)	0.9981

^a^*p*-values determined by Mann–Whitney Test.

^b^Yes versus No only.

^c^Anthracycline + taxanes vs. Other only.

^d^*p*-values determined by Fisher’s Exact Test unless otherwise specified.

### Mutation frequencies in genes and pathways

**Fig. 1:**
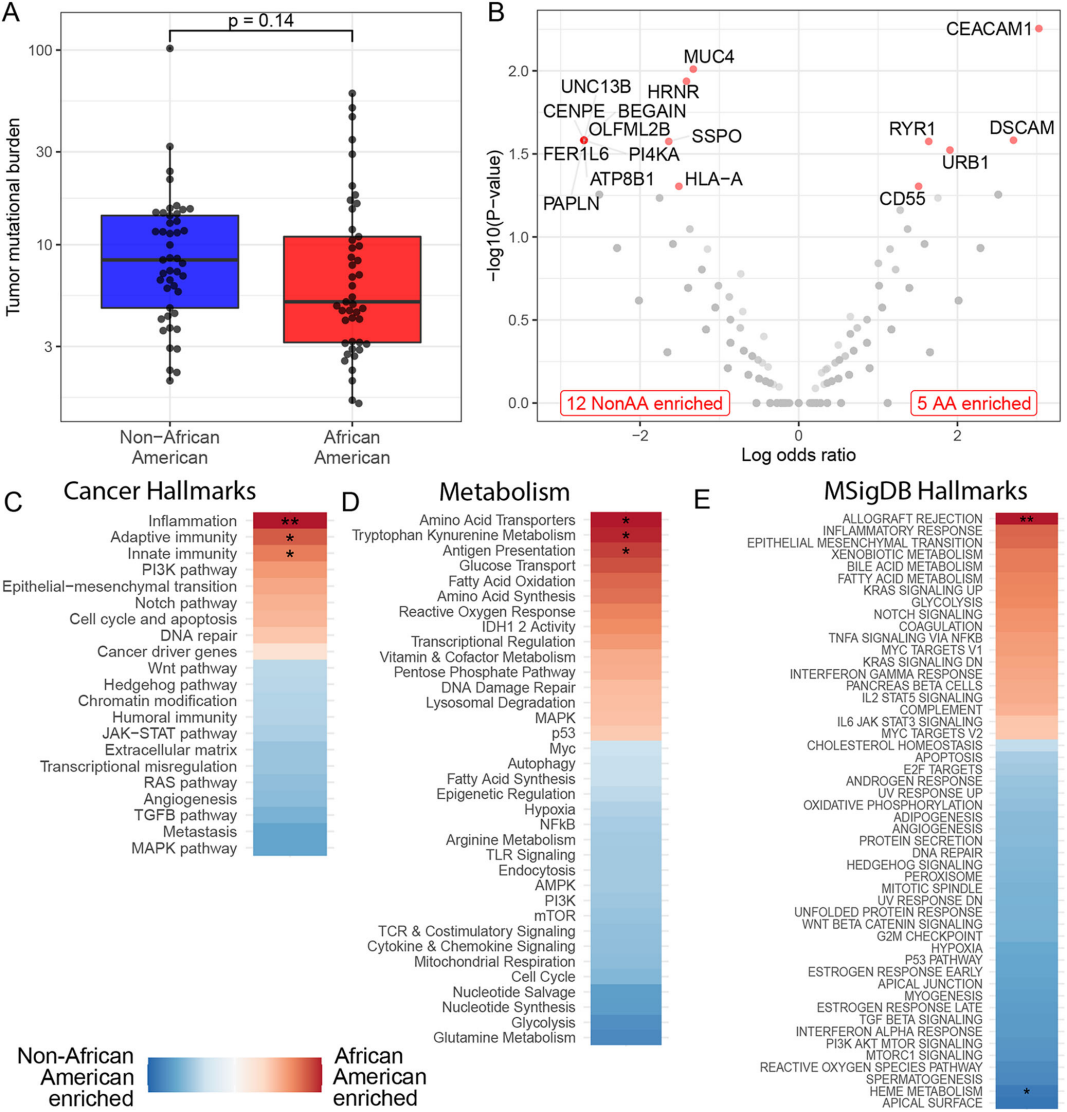
Genes and pathways affected by somatic mutations in Non-African American (NonAA) and African American (AA) TNBC patients. **A** Tumor mutation burden with median (center line) and standard deviation. **B** Volcano plot of differentially mutated genes. Genes with significantly different mutation frequencies are labeled and marked red. **C**–**E** Differential enrichment of mutations in pathways. Color coding shows the directionality of enrichment. Stars indicate significantly differentially affected pathways (*p < 0.05, **p < 0.01, ***p < 0.001; Mann–Whitney Test, Wald Test, Permutation Test).

There was no difference in tumor mutational burden between the two cohorts (Fig. 1A). Seventy-three percent of patients (*n* = 66/90) had ≥1 functional impact somatic mutation (Supplementary Data File Tables 1 and 2 and Supplementary Fig. 3A). Seventeen genes were significantly differentially affected by somatic mutations between the cohorts (*p* < 0.05; Fisher’s Exact Test) without correction for multiple testing). Twelve genes were more frequently mutated in NonAA patients and 5 genes were more frequently mutated in AA patients (Fig. 1B). Three of those genes (HLA-A; CEACAM1; CD55) were involved in immune functions. HLA-A was more frequently mutated in NonAA samples. CEACAM1 and CD55 were more frequently mutated in AA samples (Supplementary Data File Tables 1 and 2; Supplementary Fig. 3A). TCGA data from TNBC patients partially validated these observations (Supplementary Data File Table 3; Supplementary Fig. 4A, C).

11,887 somatic mutations were detected and mapped to pathways (Fig. 1C–E). Inflammation, immunity (adaptive; innate), antigen presentation, and allograft rejection pathways were significantly more affected by mutations in AA samples compared to NonAA. Among metabolic pathways, amino acid transporters and tryptophan kynurenine metabolism were more frequently affected by mutations in AA samples. Supplementary Fig. 3B–I show the leading-edge genes whose mutations drive the findings in the mutated pathways. While individual mutations were not recurrent, mutations affected chemokines (CCR7, CCR1, CXCL1), metabolism genes (IDO1), antigen presentation genes (HLA-DRA, HLA-DOA), immune checkpoint genes (PD1, CTLA4), and key immune cell genes (B cells (CD79A), T cells (CD4, CD8), macrophages (NLRP3) (Supplementary Fig. 3C–I). In NonAA patients, only genes assigned to heme metabolism pathway were more frequently mutated. A closer look at the leading-edge genes in the heme metabolism pathway revealed many genes that are relevant for cancer biology including antigen processing (CTSE), cell adhesion (ICAM4), TLR signaling (MFHAS1), neutrophil chemotaxis (MPP1), metabolism (ACSL6, PC, HAGH), cytokine production/regulation (TMEM9B, USP15, RIOK3), and apoptosis (BNIP3L, HTATIP2) (Supplementary Fig. 3B).

### Differentially expressed genes and pathways

**Fig. 2:**
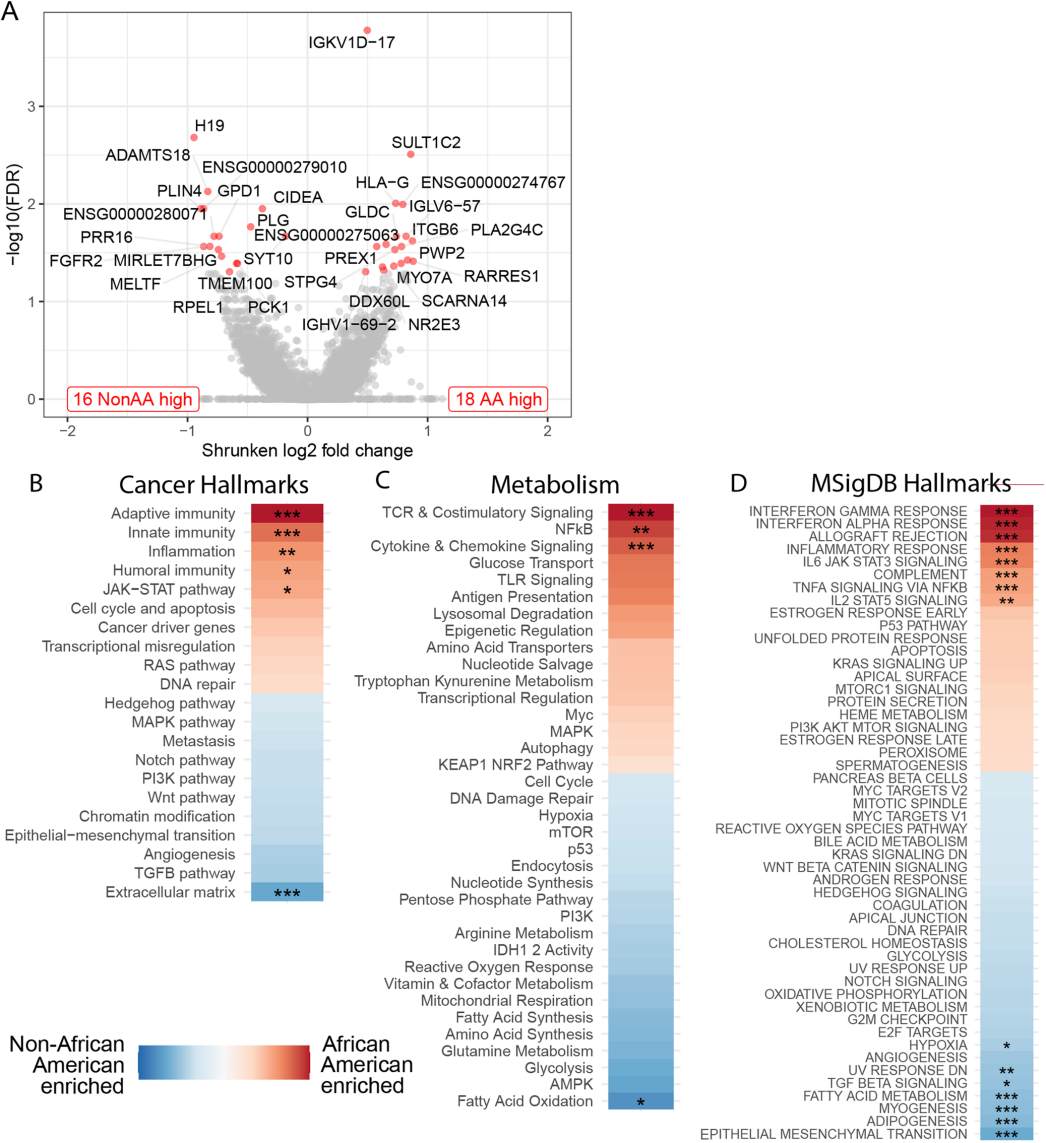
Differentially expressed genes and pathways in Non-African American (NonAA) and African American (AA) TNBC patients. **A** Volcano plot of differentially expressed genes (FDR < 0.05 and |log2 fold change | > 1 | )) are labeled and marked red. The number of up-regulated genes are shown in bottom corners. **B**–**D** Gene set enrichment analysis of Nanostring hallmarks of cancer gene set (**B**), Nanostring metabolic pathways (**C**), and hallmark pathways from MSigDB database (**D**). Color coding shows the directionality of enrichment. Stars indicate significant results (*adjusted *p* < 0.05, **adjusted *p* < 0.01, ***adjusted *p* < 0.001; Wald Test, Permutation Test).

Thirty-three genes had significant differential expression. Eighteen genes were more highly expressed in AA samples (Fig. 2A, Supplementary Data File Table 4, Supplementary Fig. 5A) including genes involved with B cell function (IGKV1D-17, IGLV6–57, IGHV1-69-2), neutrophil and cell migration (PREX1), cell adhesion (ITGB6), cell motility (MYO7A), metabolism (SULT1C2, GLDC, PLA2G4C), and immune tolerance (HLA-G). Sixteen genes were more highly expressed in NonAA samples (Fig. 2A, Supplementary Data File Table 4, Supplementary Fig. 5A) including genes linked to blood clotting (PLG), cellular iron uptake (MELTF), regulation of mammary gland lipid secretion (CIDEA, PLIN4), metabolism (GPD1, PCK1, RPEL1), and cell growth and differentiation (FGFR2).

Gene set enrichment analysis showed multiple immune-related pathways enriched in AA samples including adaptive, innate, and humoral immunity; inflammation; JAK-STAT; NFkB; cytokine signaling; T cell receptor (TCR) & costimulatory signaling; IFN responses (gamma; alpha); allograft rejection; IL6/TNFα/IL2 signaling and complement pathways (Fig. 2B–D). In NonAA samples, epithelial–mesenchymal transition, angiogenesis, adipogenesis, myogenesis, fatty acid metabolism, TGFβ signaling pathways, UV-response, and hypoxia pathways were overexpressed. Leading-edge gene plots showing the top-ranked genes driving the enrichment analysis results are shown in Supplementary Fig. 5B, C. For each of the 17 genes that were significantly differentially affected by somatic mutations between the cohorts, we found that the relationship between gene expression (i.e., lower or higher expression) and mutation status varied from gene to gene and across cohorts (Supplementary Data File Table 5).

**Fig. 3:**
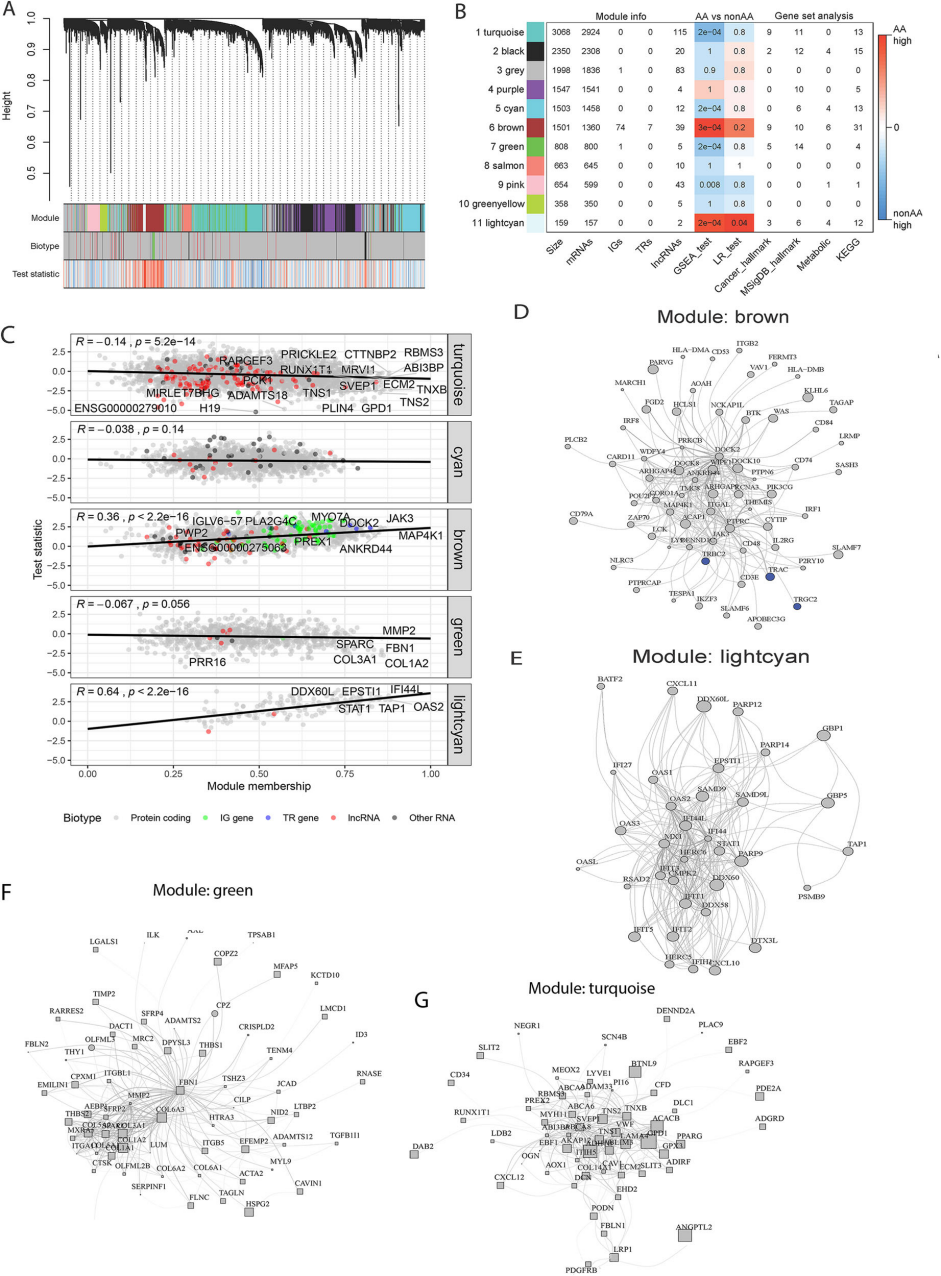
Gene co-expression network analysis. **A** Cluster dendrogram highlighting searching for gene co-expression modules. Final 11 modules are colored turquoise, black, gray, purple, cyan, brown, green, salmon, pink, greenyellow, and lightcyan. Biotype denote protein-coding genes, IG genes, TR genes, lncRNAs, or other RNAs. Test statistic is from differential analysis using DESeq package. **B** Modules description. First four columns show the module size and the number of genes categorized by biotype. Next two columns show p-values from statistical test comparing expression in NonAA vs AA patients. Last four columns show the number of enriched pathways in gene set analysis. **C** Association between module membership (correlation with module eigenvalue) and test statistic from DESEq2. Hub genes are those with highest value of module membership. **D**–**G** Topology layouts of highest module membership genes in each module. Nodes are colored by biotype. Size of the nodes is proportional to DESEq2 test statistic. Node shapes represent directionality of expression change (circle means higher expression in AA, while square means higher expression in NonAA cohort).

**Fig. 4:**
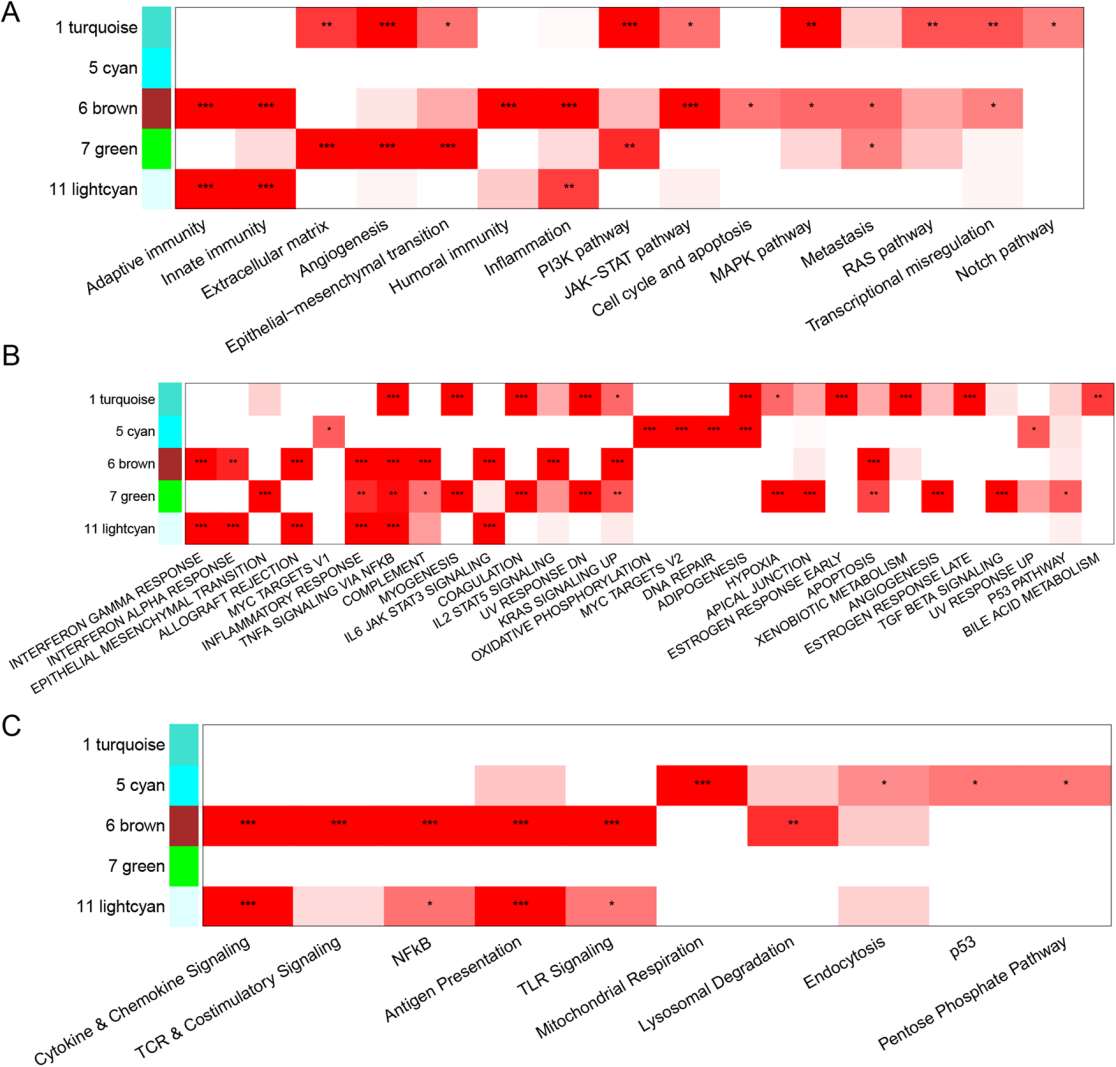
Pathways significantly enriched in the co-expression modules that were associated with African American (AA) (modules 6 and 11) or (Non-African American (NonAA) modules 1,5, 7) cancers. **A** Nanostring cancer hallmarks, **B** MSigDB Hallmarks, and **C** Nanostring metabolism (*p < 0.05, **p < 0.01, ***p < 0.001; Permutation Test).

Unsupervised clustering of 14,609 genes revealed 11 co-expression modules indicated by different colors in Fig. 3A and Supplementary Fig. 6. Three modules (turquoise, cyan, and green) were significantly more highly expressed in NonAA patients (*p* < 0.001; Permutation Test) and two modules (brown and light cyan) in AA patients (*p* < 0.001; Permutation Test) (Fig. 3B). The association between module membership, a measure of high gene connectivity within a module, and test statistic used for finding differentially expressed genes was negative in the turquoise (*p* = 8.2e–14; Permutation Test) and green (*p* = 0.056; Permutation Test) modules and positive in the brown (*p* < 2.2e–16; Permutation Test) and light cyan (*p* < 2.2e–16; Permutation Test) modules (Fig. 3C–G). Associated with the AA cohort, genes from the brown and light cyan modules were enriched in pathways that impacted the function of the immune system (Fig. 4). Associated with the NonAA cohort, genes in the turquoise and green modules were both enriched in pathways that impacted the function of the cancer cells (Fig. 4). Additionally, genes in the cyan module were enriched in pathways that affected cell metabolism (Fig. 4). This unbiased co-expression analysis was consistent with the gene set enrichment results indicating higher immune and inflammatory gene expression in AA compared to NonAA TNBC. TCGA data from TNBC patients partially validated this data with significantly differential expression of PLA2G4C, SULT1C2, PWP2, and FGFR2 genes and enrichment of genes in the adaptive immunity, interferon response (gamma; alpha), and JAK-STAT signaling pathways (Supplementary Fig. 4B, D).

### sTILs scores, PD-L1 protein expression, TIDE, and CIBERSORT

The percent of sTILs was significantly higher in AA compared to NonAA samples (*p* = 0.0302; Mann–Whitney Test) (Supplementary Fig. 7A). PD-L1 percent positivity was assessed with two clinically used antibodies, SP142 and SP263. The SP142 assay showed significantly higher PD-L1 expression in the AA cohort (*p* = 0.02; Mann–Whitney Test) (Supplementary Fig. 7B). The SP263 assay showed a similar trend but the difference has not reached statistical significance (Supplementary Fig. 8A). Patients with a higher level of sTILs and/or PD-L1 positivity had more favorable recurrence-free survival outcomes regardless of self-reported race (Supplementary Fig. 2). When the AA and NonAA cohorts were divided into PD-L1 positive and negative or sTILs high (≥30%) and low (<30%) biomarker groups, respectively, the PD-L1 positive and sTILs high groups showed a strong trend for better recurrence-free survival in NonAA patients. However, in AA patients, recurrence-free survival benefit was weakly associated only with PD-L1 positivity (Supplementary Fig. 2). This data suggests that high sTILs or PD-L1 expression is less of a good prognostic marker in AA compared to NonAA.

We used TIDE and CIBERSORTx analysis to quantify immune functions and immune cell types in the tumors. We found no difference in the immune dysfunction, microsatellite instability (MSI), myeloid-derived suppressor cells (MDSC), and cancer-associated fibroblasts (CAF) scores, or in mRNA levels of CD274 (PD-L1) and CD8 (Supplementary Fig. 8B–G). On the other hand, the tumor-associated macrophage TAM M2 score (*p* = 0.0004; Mann–Whitney Test) and the Immune Exclusion score (*p* = 0.004; Mann–Whitney Test) were significantly higher in NonAA patients (Supplementary Fig. 7C, F). The “immune inflamed” signature (Merck18) (*p* = 0.0170; Mann–Whitney Test) and the IFNG signature (*p* = 0.0563; Mann–Whitney Test) scores were higher in AA samples (Supplementary Fig. 7D, E). Similar to our previous TCGA analysis^14^, CIBERSORTx immune cell types were not different between the two cohorts (Supplementary Figs. 9 and 10).

**Fig. 5:**
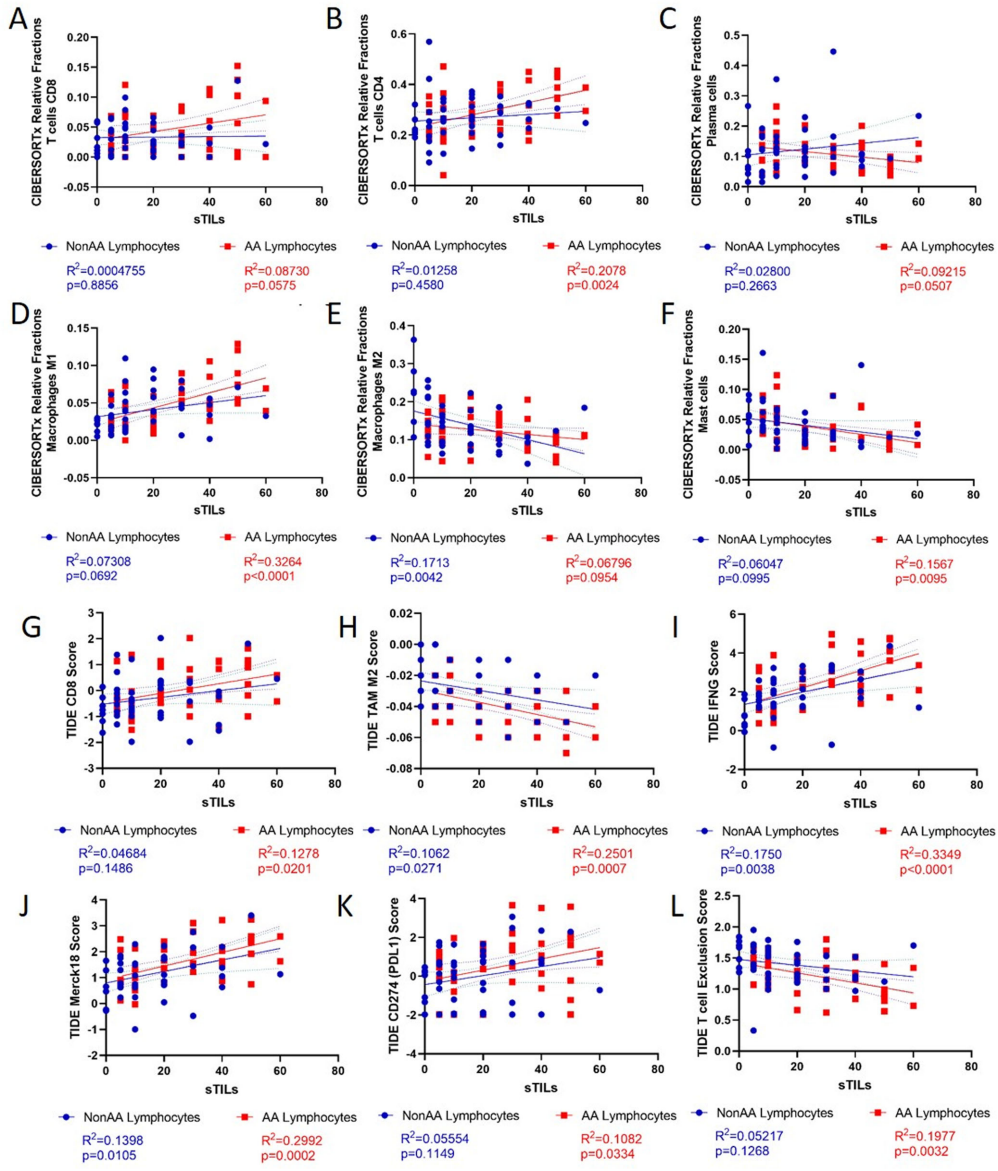
Correlations between histological stromal TILs (sTILs) and key immune signatures in TNBC in Non-African American (NonAA) and African American (AA) patients. **A**–**F** Deconvoluted CIBERSORTx immune cell fractions or **G**–**L** TIDE signature scores. Blue = NonAA. Red = AA. *p*-values from linear regression.

We identified correlations between sTILs and key immune signatures from CIBERSORTx and TIDE (Fig. 5, Supplementary Figs. 11 and 12). In AA samples, sTILs positively correlated with CD4 and CD8 T cells, M1 macrophages, CD274 (PD-L1) score, NK cells, MSI score, and immunotherapy response predictive signatures (Merck18; IFNG), and negatively correlated with plasma cells, mast cells, TAM M2 score, CAF score, and T cell Exclusion score. In NonAA samples, sTILs positively correlated with immunotherapy response predictive signatures (Merck18; IFNG), and negatively correlated with M2 macrophages and TAM M2 score (Fig. 5, Supplementary Figs. 11 and 12). There were no correlations with other cell type scores (Supplementary Fig. 12). CIBERSORTx and TIDE shared two similar populations, CD8 and M2 Macrophages. The CIBERSORTx and TIDE CD8 positive populations had a positive correlation. However, the TIDE TAM M2 score and CIBERSORTx Macrophage M2 showed no correlation which is possibly due to differences in the composition of these signatures and methods (Supplementary Fig. 13).

## Discussion

The self-identified AA and NonAA TNBC patients in this study were evenly matched for clinical and pathological variables. However, we did find that AA patients had more comorbidities (obesity; hypertension; type 2 diabetes mellitus), received less aggressive therapies (46% of AA had taxanes/anthracycline combination compared to 71% of NonAA patients), experienced higher recurrence rates (33.3% vs. 14.3%), and worse recurrence free survival. Differences in recurrence rates and mortality between AA and NonAA patients have been repeatedly observed in the literature and multiple factors contribute to it. At population level, delayed access to care and to screening increases recurrence rates, in institutional cohorts, like ours, higher co-morbidity rates and greater difficulty to complete prescribed therapies could explain much of the difference in outcome in stage matched cases. We also note that survival comparisons in this study are limited because of the small sample size and highly selected cases.

WES and RNAseq results revealed no differences in somatic tumor mutation burden and only small differences in mutation frequencies and expressions of genes. Among the genes more frequently mutated in AA samples, CEACAM1 (NEO-201; NCT03476681) and CD55 represent potential therapeutic targets. Interactions between CEACAM5/6 on tumor cells and CEACAM1 on immune cells inhibit immune-mediated cytotoxicity^15^. CD55 is a complement binding protein that inhibits complement mediated cell lysis^16,17^. We previously reported that CD55 expression is higher in metastatic versus primary breast cancers and may contribute to the immune attenuated microenvironment of metastatic lesions^18^. TCGA partially validated the observations in the Yale Cohort, however several of the genes more frequently mutated in our cohorts were not measured in TCGA. This could be due to processing differences including lower sequencing coverage in the TCGA, differences in variant calling methods between studies, differences in source material since we worked with FFPE while TCGA used frozen tissue, and small sample sizes in both datasets coupled with substantial and inherent inter-tumor variability^19^. A limitation of our analysis is that we cannot determine from the single-nucleotide sequence alterations if the effect is enhancement or reduction of the normal function of these molecules.

A more consistent and previously unrecognized phenomenon emerged when we analyzed somatic mutations at the pathway level. Pathways impacting the immune system and metabolism were more frequently affected by mutations in AA patients. The affected genes varied from cancer to cancer, were not recurrent, and therefore missed at the gene level.

Among the genes more highly expressed in NonAA patients, FGFR2 and CD228 represent therapeutic targets. FGFR inhibitors have been tested in breast cancer with modest success so far^20,21^. CD228 is an iron and zinc transporter that is expressed in many cells types^22–25^, and an anti-CD228-MMAE antibody–drug conjugate is in clinical trials (NCT04042480). Among the genes more highly expressed in AA patients, drugs against HLA-G (TTX-80; NCT04485013) and ITGB6 (SGN-B6A-MMAE antibody–drug conjugate; NCT04389632) are in clinical trials. HLA-G is an MHC-I molecule expressed on placental cells and cancer cells and potentially mediates immune tolerance^26–30^. Interestingly the expression of this gene is enriched in metastatic lesions versus primary breast cancers^18^. ITGB6 is a cell adhesion receptor that binds to ligands like fibronectin and TGFB1. It is upregulated in cancers including breast cancer and is associated with increased proliferation, migration, and invasion of cancer cells as well as poor prognosis^31,32^. Whether any of these drugs will show differential activity by self-identified race is unclear.

When we examined pathway-level differential expression, we observed higher levels of expression of immune pathways in AA TNBC. Many of these pathways were also more frequently affected by somatic mutations. In NonAA patients, higher levels of expression were observed in pathways related to cancer biology and metabolism. Co-expression modules in unsupervised hierarchical clustering validated these findings. In AA samples, assessment of immune cell types and function through TIDE analysis indicated lower tumor-associated macrophage M2, tumor immune dysfunction and exclusion prediction scores, and higher “immune inflamed” and interferon-gamma signatures, which have previously been shown to predict response to immunotherapy.

The higher immune and inflammatory gene expression in AA compared to NonAA TNBC was corroborated by higher histologic sTILs scores and more frequent PD-L1 protein expression. In AA samples, sTILs positively correlated with a broader array of immune cells that promote anti-tumor response and negatively correlated with cancer-associated fibroblasts (CAF) suggesting a role for pro-tumor response which was not identified in NonAA samples. Interestingly, in NonAA samples, sTILs negatively correlated with M2 macrophages and TAM M2 score suggesting a possibly different role for macrophages in tumor promotion in these patient populations.

In our cohort, AAs had significantly higher comorbidities which may result in chronic systemic inflammation that might prime the immune system to be more reactive and therefore produce higher levels of sTILs and PD-L1 positivity in the tumor microenvironment. These comorbidities may also change the composition of sTILs which may explain why we found that CD4 T cells positively correlated with sTILs in AA samples and not NonAA samples. It is possible that although the immune response seems to be more prominent in AA TNBC tumor microenvironment, the anti-cancer activity could be attenuated in the context of systemic low-level inflammation. However, an important limitation of this study is that we were not measuring systemic inflammatory markers.

Our findings raise the possibility that immune checkpoint inhibitors and other immunotherapy drugs might have differential efficacy in AA patients. The IMpassion130 (NCT02425891) clinical trial of atezolizumab with nab paclitaxel as first line chemotherapy for metastatic TNBC had an enrollment of 6.0% AA women and 68.0% NonAA women. The improvement in survival was most dramatic in PD-L1 positive AA patients (*n* = 21) resulting in a hazard ratio (HR) of 0.07 [95.0% CI 0.01–0.55] compared to the HR 0.71 [95.0% CI 0.52–0.98] in NonAA patients^33^. The neoadjuvant IMpassion031 trial also showed nominally higher absolute increase pCR rate (18.0% versus 13.0%) in AA patients (*n* = 24) compared to Caucasian patients (n = 210)^34^. However, the very small number of AA patients in these trials make these comparisons very preliminary. In addition, caution should be exercised as an enhanced immune infiltration and inflammatory microenvironment in AA TNBC may increase immunotherapy-induced toxicity in number and/or severity.

Limitations of our approach include inherent inability to account for all variables that impact inflammation in breast tissue, although in our analysis we adjusted comparisons for obesity, other factors such as diet, level of stress, or concomitant medications could not be considered. In addition, we used self-identified race found in medical records to assign NonAA and AA categories, but we recognize patients come from a wide range of countries, ethnicities, cultures, and environments which can impact immune characteristics. However, our overall observations are consistent with a more “inflamed” breast tumor microenvironment in AA patients that was also observed by others^35^. These correlative studies cannot answer the question why AA patients have a more immune-active breast tumor microenvironment. Socioeconomic factors contribute to rates of obesity, as well as frequency and severity of co-morbid illnesses that can affect stress level which in turn could affect the immune system. Correlation between socioeconomic status, comorbidities, and poorer cancer survival is observed in all racial groups in the USA. The impact on comorbidities on outcome have also been shown in other countries. A recent study in 445 NonAA patients in France and Belgium showed that high sTILs were associated with pCR, increased event-free survival, and overall survival in lean patients but not in patients with BMI > 25^36^. These findings are important as they suggest that comorbidities should be considered for de-escalation trials that use sTILs for patient stratification. In addition, a recent study utilizing the TCGA database^37^ and a review on genetic diversity in Africa^38^ have suggested that phenotypic and genetic variation in the immune system is heritable and adaptive. In our study, we found no differences in functional impact germline mutation frequencies in cancer-relevant genes between NonAA and AA cases. We only observed differences at somatic mutation and gene expression levels. In addition, although average inflammatory signatures were higher in AA patients, many NonAA patients also had high inflammatory gene signatures. Group-level analysis is helpful in uncovering potential population differences, but individual patient immune profiles are required to treat the patient regardless of their ancestry.

In conclusion, there is greater immune infiltration and inflammation in TNBC of AA patients and greater alterations and expression of genes that directly impact cancer cells and cellular metabolism in NonAA patients. We also identified several therapeutic targets that are currently in clinical development that could potentially be tested in TNBC patients. Studies such as ours highlight the need to ensure that all ancestries and ethnicities are appropriately represented in clinical research to learn about potential differences in treatment response.

## Methods

### Patient population and samples

Formalin-fixed paraffin-embedded (FFPE) pretreatment surgically resected cancer and paired normal tissues, and corresponding electronic medical record clinical information were collected for 56 NonAA and 54 AA Stage I-III TNBC at Yale Cancer Center between 2000 and 2017. The two cohorts were selected to match for age at diagnosis, tumor size, nodal status, histologic grade, and year of diagnosis (Table 1). The sample size reflects the maximum number of available cases that met the selection criteria. This sample size provides an 80.0% power to detect an effect size of 0.53 and a 90.0% power to detect an effect size of 0.6 when comparing distributions of the molecular variables. The number of cases available for the different types of molecular analyses and sample disposition are shown on Supplementary Fig. 1. This study was approved by the Yale Human Investigation Committee.

### Isolation of DNA and RNA from TNBC and normal tissue

DNA and RNA isolation and sequencing were performed at the Yale Center for Genome Analysis^39^. Briefly, for each case, ten 10 µm FFPE curls were obtained, four curls were used for somatic and germline DNA extraction and two curls for RNA extraction. DNA and RNA were isolated using the QIAamp DNA FFPE Tissue Kit (QIAGEN, cat. no. 56404) and RNeasy FFPE Kit (QIAGEN, cat. no. 75304), respectively, following the manufacturer’s instructions. Quality and concentration of isolated DNA and RNA was tested on the Agilent 2100 Bioanalyzer system. DNA and RNA sequencing were performed at the Yale Center for Genome Analysis.

### Whole exome sequencing (WES)

Genomic DNA (1 μg) of tumor and matching normal tissue was sheared to a mean fragment length of 220 bp using the Covaris E210 instrument, purified by Magnetic AMPure XP Beads (Beckman Coulter), and labeled with 6-base barcode during PCR amplification^39^. Exomes were captured using the IDT xGen Exome Research Panel v1.0. Libraries were sequenced on Illumina HS4000 Illumina instrument using 100-base pair paired-end reads by multiplexing four tumor samples per lane to sequence to a median coverage of 142× for tumor samples and 66× for normal samples.

### Somatic mutation and pathway analysis

Raw sequencing data was generated by the Hiseq400 in the Yale Center for Genome Analysis. Sequencing data of tumor and matched normal samples were mapped to the human reference genome vGRCh38 using Burrows–Wheeler Aligner (v0.7.15)^40^. PCR duplicates were marked in the aligned BAM file using picard (v 2.17.11, http://broadinstitute.github.io/picard/). Indelrealigner and RealignerTargetCreator kits of GATK (v3.4)^41^ were used to adjust the alignment of indel regions.

Mutect was used (v.1.1.4)^42^ to identify somatic single nucleotide variants (SNV). IndelGenotyper (36.3336) of GATK (v3.4) was used for somatic indel calling. Variants that were considered likely to be germline in dbSNP were excluded. The following quality metrics were then applied to the mutation data to select usable cases for downstream analyses: number of reads, mean coverage, and PCR duplication rate in normal and cancer samples. Cases with lower quality criteria than others, based on Tukey criterion, for at least one of the metrics described above were excluded from all mutation analyses (45 AA and 45 NonAA samples remain). To control for the false positive rate of germline variants calling, low-quality variants were removed. Since distribution of coverage in normal and tumor samples was different (higher in tumor), we removed variants that had a coverage rate too low based on Tukey criterion, in either normal or cancer samples leaving 29,322 unique variants.

Missense, nonsense, frameshifting, splice-site altering single-nucleotide changes, and indels were selected as functional impact somatic mutations. A gene was considered mutated if at least one functional impact mutation was found within its region. Tumor mutational burden (TMB) was defined as the number of somatic mutations per sequenced megabase of exome region. The mutation frequency of a gene was defined as the ratio of the number of cases with ≥1 functional impact somatic mutation per total number of cases in AA or NonAA. For genomic data in TCGA, somatic mutations of 50 AA and 90 NonAA TNBC cases were obtained from the MC3 dataset^43^.

Fisher’s exact test was used to find differentially mutated genes between the two cohorts. Log odds ratios with a Haldane–Anscombe correction were calculated to quantify the magnitude of mutation rate difference. Pathway enrichment analysis was performed using the fgsea package in R^44^ on pathways included in the Nanostring hallmarks of cancer gene set (*n* = 21), Nanostring metabolic pathways (*n* = 36), and hallmark pathways from MSigDB database^45^ (*n* = 50). Log odds ratio was used as a gene rank value in fgsea.

### RNA sequencing (RNAseq)

RNA sequencing libraries were prepared from 1 µg of RNA using PolyA selection with oligo-dT beads, followed by random priming using the Illumina TruSeq Stranded Total RNA kit. Samples were sequenced with a target coverage of 50 million reads, paired-end, using the Illumina NovaSeq 6000 S4 platform. Raw sequencing reads were assessed for quality using FastqQC v0.11.5^46^ and adapter sequences were removed using Trimmomatic v0.36^47^. During pre-processing, paired-end RNA fastq files were aligned to the reference genome Hg38 using the STAR method with two-pass mode and default parameters^48^. Gene expression was quantified using RSEM v1.3.0^49^. The following quality metrics were then applied to the pre-processed RNA-sequencing data to select usable cases for downstream analyses: number of paired reads, percent uniquely mapped reads, percent counts within exons, percent counts in protein coding genes, and average distance of coverage distribution. Cases with lower quality criteria than others and fewer than 5 million counts in exons were excluded from all gene expression analyses. The ESTIMATE algorithm was used on pre-processed RNA sequencing data to compare the purity of tumor tissue in each sample^50^. Purity for NonAA was 1125.89 with a range of −1696.49 to 4007.213 and for AA it was 1350.19 with a range of −475.93 to 3789.79 based on gene expression.

### Gene expression and pathway enrichment analysis

To find the genes that were differentially expressed (FDR < 0.05 and log2 fold change | >1 | ) between NonAA and AA, we used the DESEq2 package in R^51^. Two covariates were added to the model: (i) stromal score obtained by ESTIMATE R package to normalize for different tumor purity between samples; and (ii) patient obesity as a significant clinical variable. For expression data from TCGA, 19,885 expressed genes from RNAseq were obtained from 50 AA and 92 NonAA TNBC cases. DESEq2 was used with stromal score as covariate. Gene-set enrichment analysis (GSEA; Permutation Test) was performed using the fgsea package in R^44^ and DESeq2 test statistics (Wald Test) as rank value.

Gene co-expression network analysis was performed using WGCNA package in R^52^. The DynamicTree Cut algorithm^53^ was used to identify the modules of clusters of highly interconnected genes. Each module is represented using a different color. Module eigengenes (MEs) were estimated as 1st principal component of expression of genes in a module and then used to merge similar modules using hierarchical clustering. To quantify module membership (MM), the biweight midcorrelation between expression profile of each gene and ME was calculated. Association between modules and NonAA or AA was evaluated using two methods: (i) GSEA test—GSEA (Permutation Test) as described above with genes annotated to each module treated as different gene sets; (ii) LR test—logistic regression model comparing expression of MEs between NonAA and AA. Pathway enrichment analysis was performed on genes in each module separately as described above. Modules of co-expressed genes were visualized using undirected graphs (igraph R package)^54^ with topological overlap measure.

### Analysis of cellular composition with TIDE and CIBERSORTx gene signatures

The Tumor Immune Dysfunction and Exclusion (TIDE) web-based tool was used to evaluate the potential of tumor immune escape using gene expression profiles including “immune inflamed” Merck18 signature score (Merck18), interferon-gamma response signature (IFNG) score, microsatellite instability (MSI) score, CD274 (PD-L1 gene), CD8 (average of CD8A + CD8B) genes, T cell dysfunction score, T cell exclusion score, myeloid-derived suppressor cell (MDSC) score, cancer-associated fibroblast (CAF) score, and tumor-associated macrophage M2 type (TAM M2) score^55–57^. To create a matrix for analysis for the TIDE tool^55,56^, RNAseq values were log transformed into log2(TPM + 1). Each gene was normalized by subtracting the average value of all patients in the cohort. Mann–Whitney test was used to determine if there were significant differences (*p* < 0.05) in TIDE gene signatures between NonAA and AA.

CIBERSORTx was performed as previously described^14^. The relative fractions of 22 immune cell sub-populations were estimated using the CIBERSORTx web-based tool, the LM22 reference gene expression signatures, and a non-log linear gene expression matrix of the cohort for analysis^58,59^. Kruskal–Wallis Test was used to determine if the medians of the immune cell sub-populations were significantly different. The mean rank of each subpopulation was compared between racial groups using Dunn’s multiple comparison test with a multiplicity adjusted *p*-value for each comparison. Significance was defined as *p* < 0.05.

### Analysis of relationships between somatic mutations and their corresponding gene expressions

To determine the relationship between gene mutations and expression, the average VST normalized expression levels of genes between individuals with mutations (affected) and those without mutations (nonaffected) were calculated for genes that had greater than 10 cases affected by functional impact mutations. The Mann–Whitney test was used to determine if there were significant differences (*p* < 0.05) in gene expression levels between affected and non-affected genes (Supplementary Data File Table 5).

### Histology

To quantify sTILs, 4 µm histology sections were deparaffinized, hematoxylin & eosin stained, digitally scanned, and scored independently by two pathologists. The percentage of sTILs were calculated as the area occupied by mononuclear inflammatory cells over the total intratumoral stromal area^60^. sTILs scores ≥30% were considered high and those <30% considered low^61^. Two additional sections were used for PD-L1 (SP263 (RTU-IVD, Catalog #740–4907); SP142 (RTU-IVD, Catalog #740-4859)) (Roche, Indianapolis, IN) protein evaluation by immunohistochemistry (IHC) using the Ventana Benchmark autostainer (Roche) following manufacturer’s instructions. Tissues with ≥1.0% immune cell PD-L1 in the tumor stroma were considered positive as scored by three independent pathologists^60,62^. Mann–Whitney test was used for sTILs scores and the Fisher’s exact test was use for PD-L1 IHC positivity to determine if there were significant (*p* < 0.05) differences between NonAA and AA.

### Statistical analysis

Statistical analyses are described in detail in the subsections. Demographic and clinical data were analyzed using R package v4.0.3 or GraphPad Prism v9.0. For Age and Follow-up time in years, interquartile range (IQR) are presented as medians with 25.0–75.0% IQRs. Categorical variables were assessed using either Chi-square or Fisher’s exact test. Continuous variables were compared using either Mann–Whitney test or Kruskal–Wallis test with Dunn’s or Benjamini–Hochberg post hoc tests for multiple testing. Kaplan–Meier plots were used to visualized regression-free survival. The somatic mutation, gene expression, gene co-expression, and all pathway analysis were analyzed using R. The histology variables (sTILs; PD-L1 IHC), TIDE, and CIBERSORT were analyzed using GraphPad Prism. For all analysis, statistical significance was defined as two-sided *p* < 0.05.

## Supplementary information

Supplemental Figures

Supplementary Data File

## Data Availability

All data associated with this study are present in this paper or Supplemental Materials. The whole exome and transcriptomic data are deposited in National Center for Biotechnology Information (NCBI) database of Genotypes and Phenotypes (dbGaP) under Bioproject #704957.
