# Supplementary material for: Tumor immune microenvironment of self-identified African American and non-African American triple negative breast cancer
Source: NPJ Breast Cancer. 2022 Jul 22;8:88. doi: 10.1038/s41523-022-00449-3 (PMC9307813; doi:10.1038/s41523-022-00449-3)
Supplement: Supplementary file 1 — Supplemental Figures [file 41523_2022_449_MOESM1_ESM.pdf]

Supplementary Figures

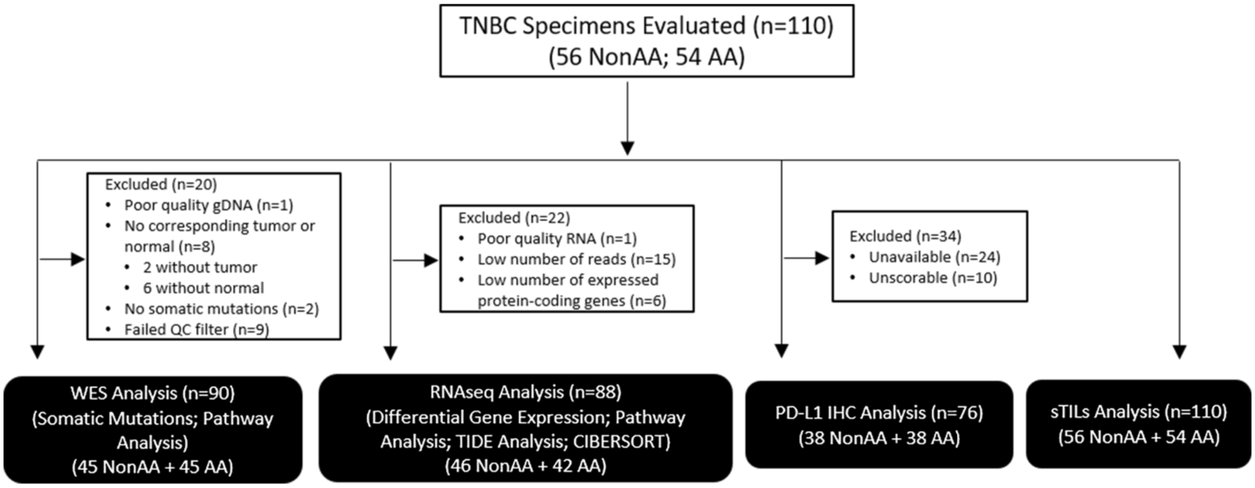

Supplementary Figure 1. Consort diagram of samples for comparison of the FFPE tumor microenvironment of TNBC Non-African Americans (NonAA) and African American (AA) patients.

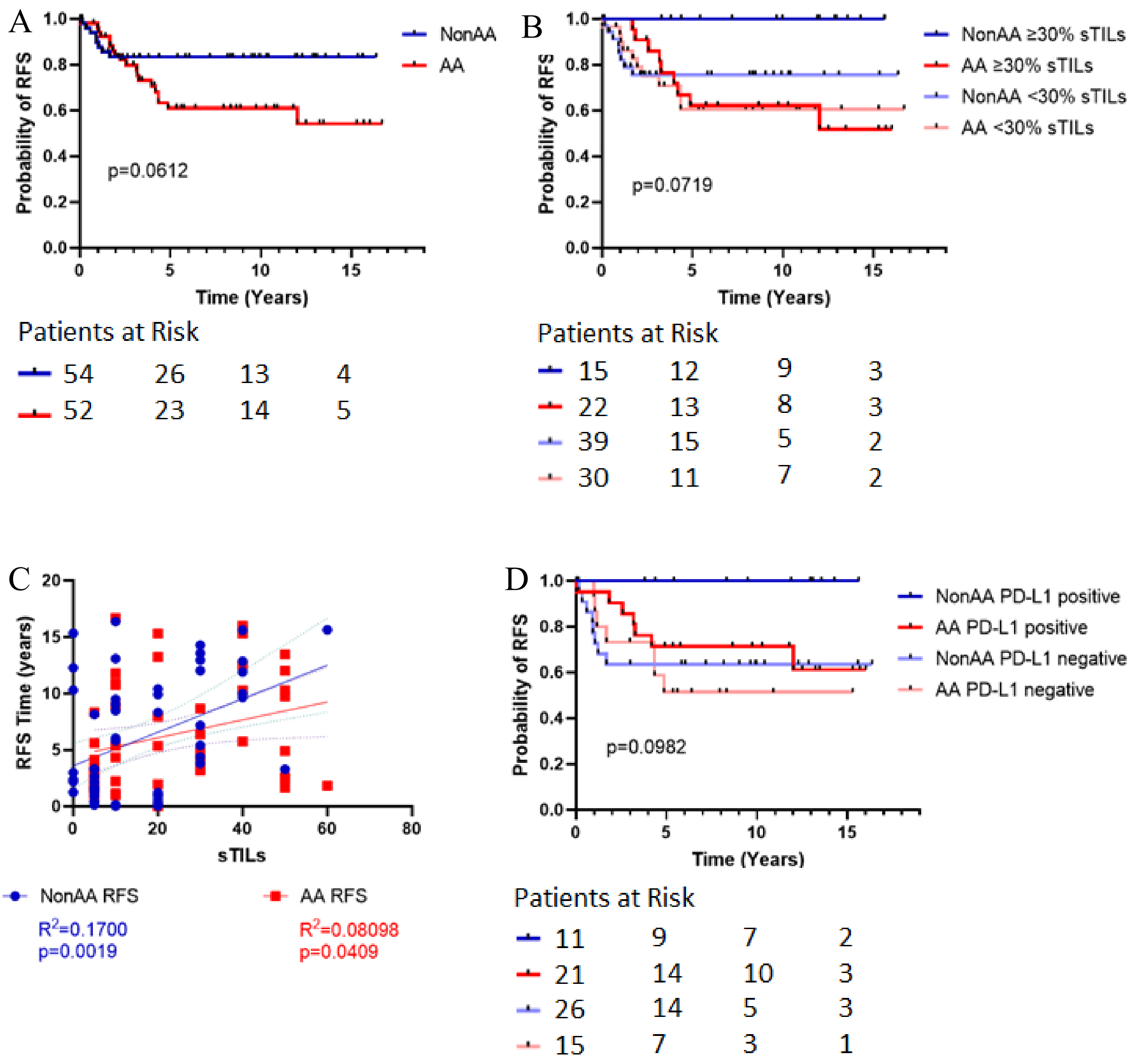

Supplementary Figure 2. Recurrence Free Survival in age and pathology matched TNBC in Non-African American (NonAA) and African American (AA) patients. (A) Recurrence Free Survival (RFS). (B) RFS stratified by sTILs scores dichotomized by ≥30% versus <30% (Park, et. al 2019 Ann Oncol [1]). (C) Correlation between sTILs and RFS. (D) RFS stratified by SP142 PD-L1 positivity (yes vs no). P-values from Mantel-Cox Test (A, B, D) or Linear Regression (C).

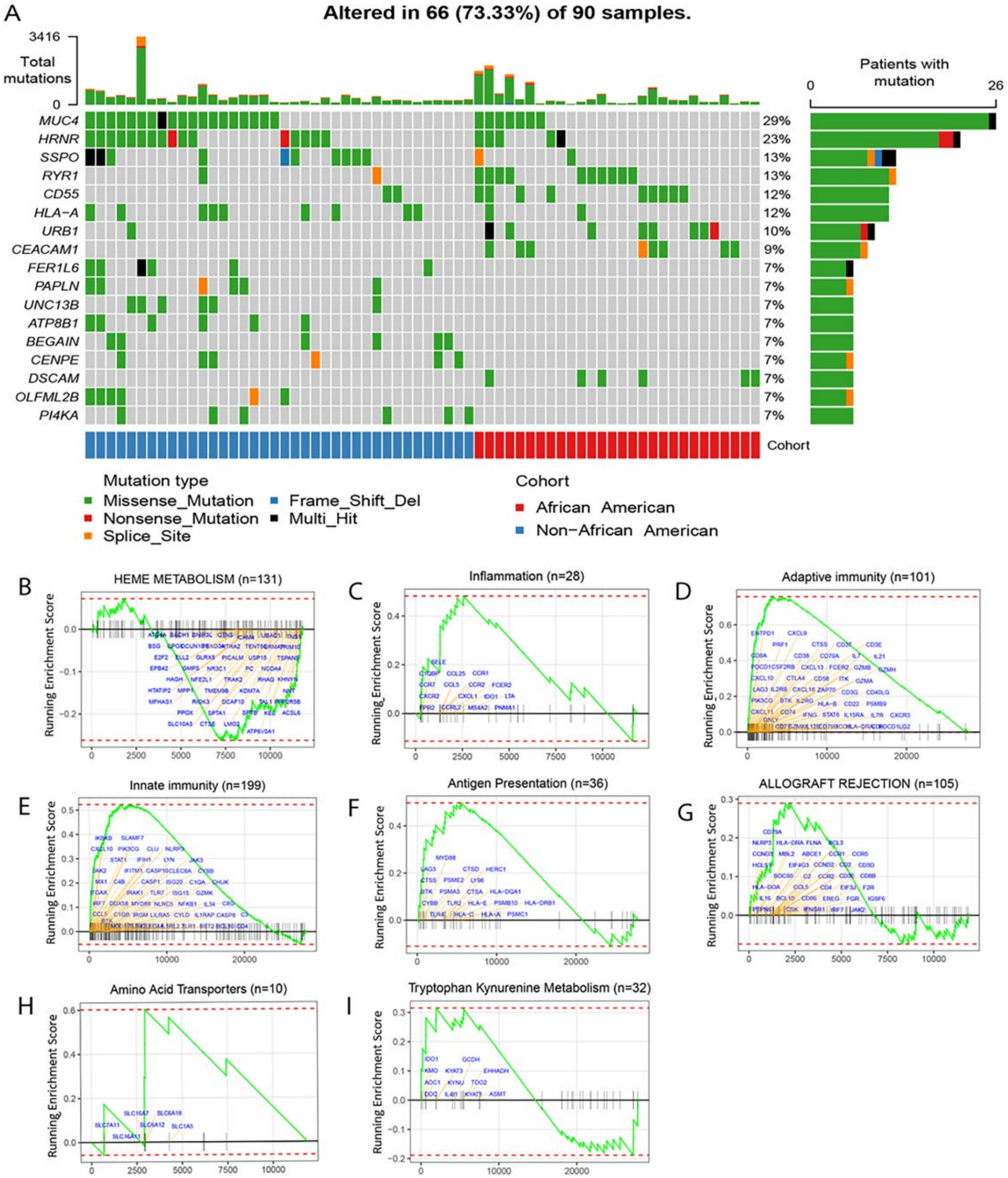

Supplementary Figure 3. **Somatic mutations in Non-African American (NonAA) and African American (AA) TNBC patients.** (A) Distribution of somatic mutations in 17

differentially mutated genes. (B-I) Leading edge genes that drive the significance of the findings in the 8 pathways from Figure 1 that were differentially affected by mutations in NonAA patients (B) and AA patients (C-I).

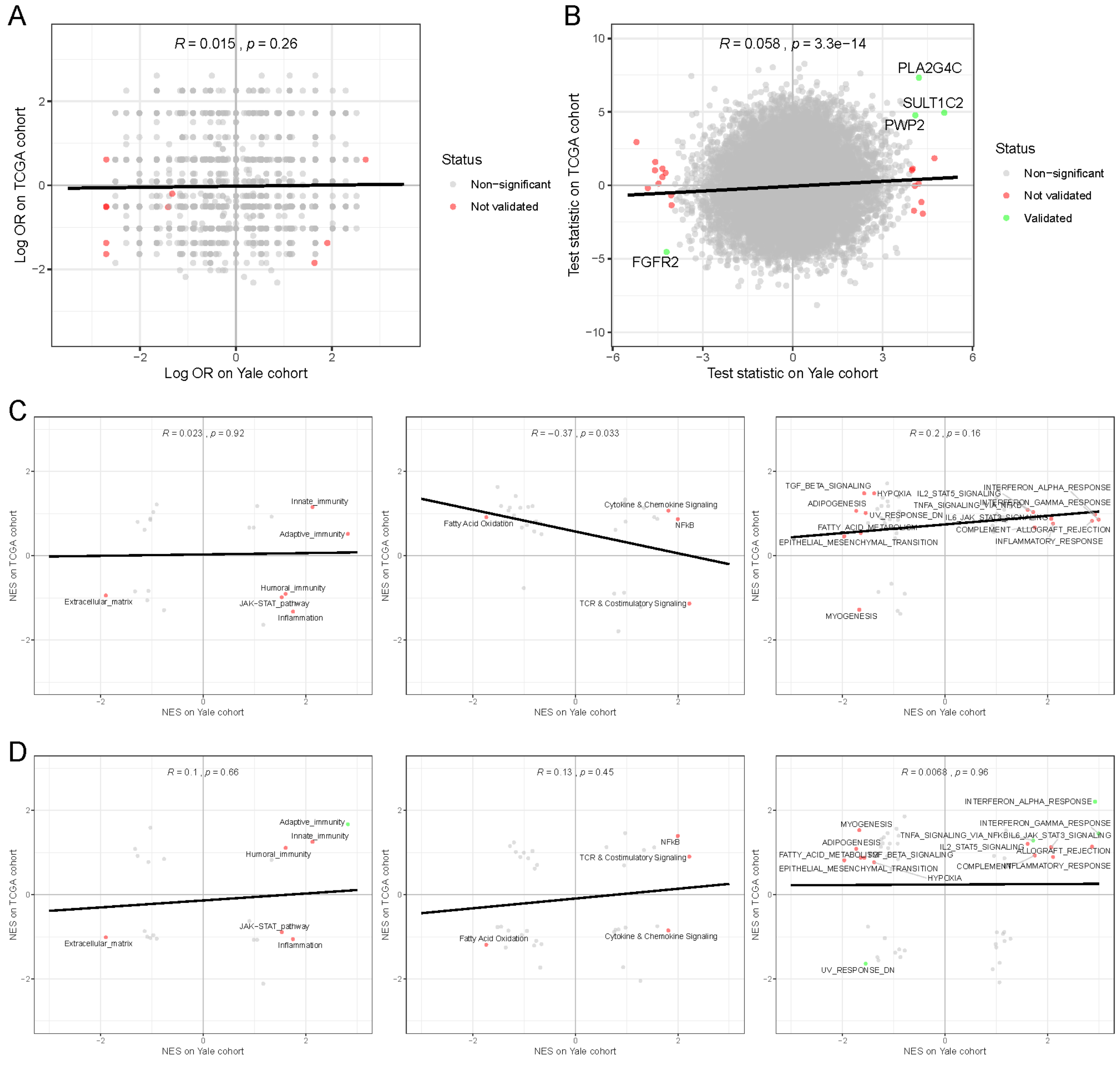

Supplementary Figure 4. Validation of somatic mutations, differentially expressed genes, and gene sets using TCGA for comparison in Non-African American (NonAA) and

African American (AA) TNBC patients. Correlation between Yale and TCGA cohorts for (A) Somatic mutations from whole exome sequencing, (B) Differentially expressed genes, and (C-D) Cancer Hallmark, Metabolic, and MSigDB hallmark gene sets, respectively from WES (C) and RNA sequencing (D) analysis. OR = odds ratio. NES = Normalized Enrichment Score.

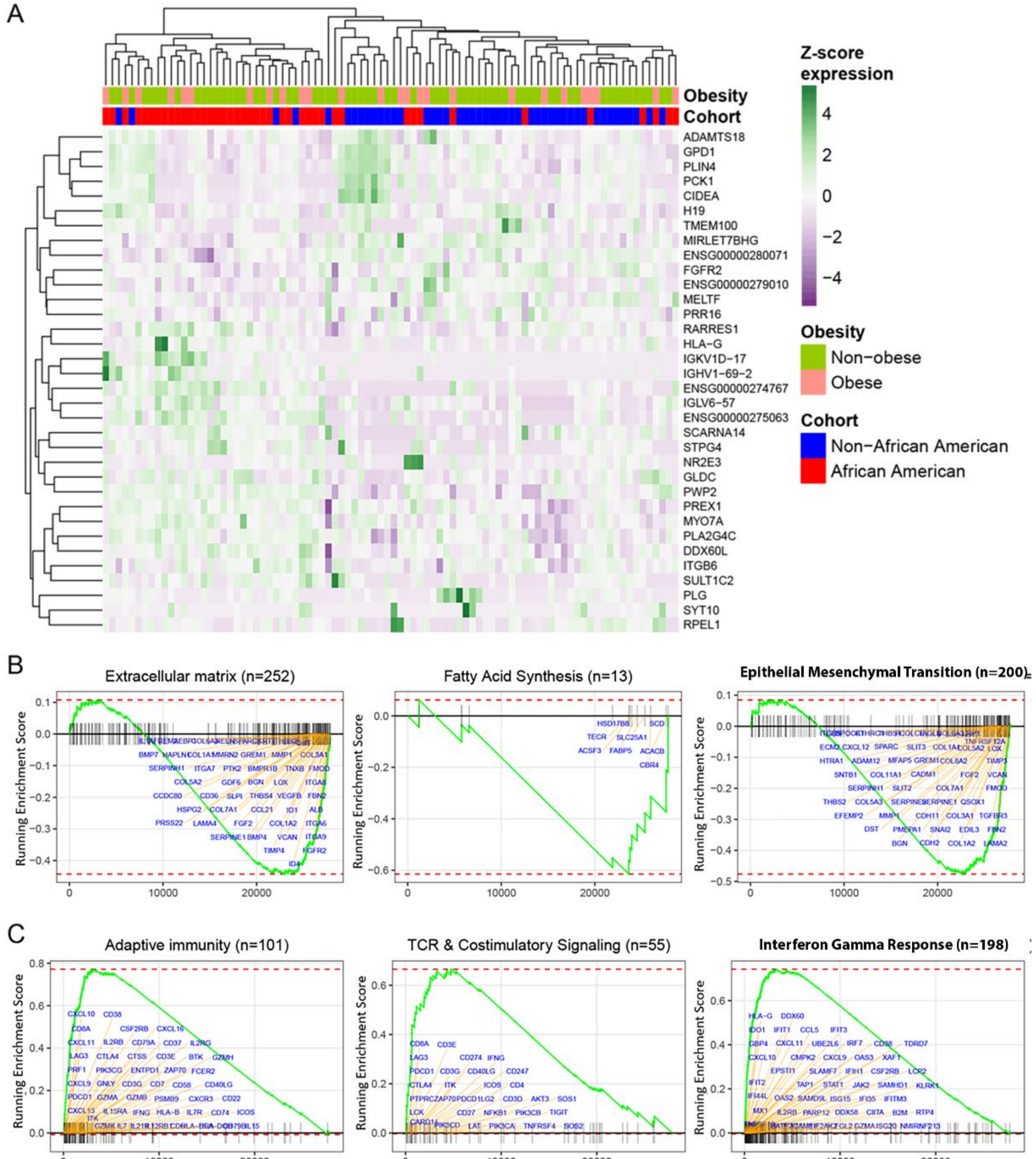

Supplementary Figure 5. **Heat map and leading-edge gene plots from pathway enrichment analysis of differentially expressed genes in Non-African American (NonAA) and African American (AA) TNBC patients.** (A) Heatmap of all significantly differentially

expressed genes. (B-C) Leading edge fraction genes for the top significantly enriched pathways in NonAA patients (B) and AA patients (C).

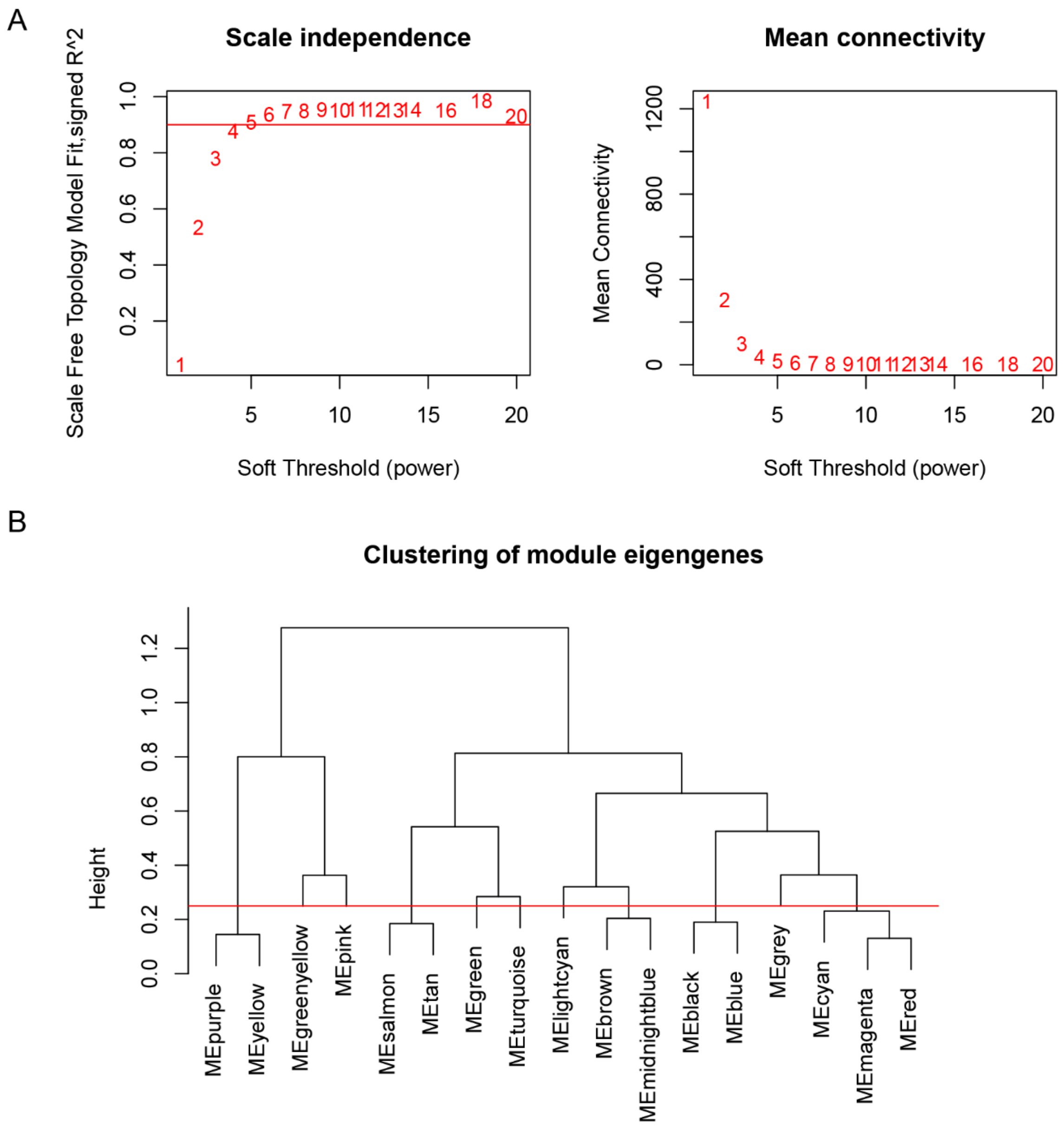

Supplementary Figure 6. Processes for determining module selection and topology in Non-African American (NonAA) and African American (AA) TNBC patients. (A) Scale Free Topology Fitting Index (determines robustness of analysis) and biological signals

(determines intramodular connectivity and prognostic significance) for finding soft threshold (power). (B) Clustering of module eigengenes to merge similar modules.

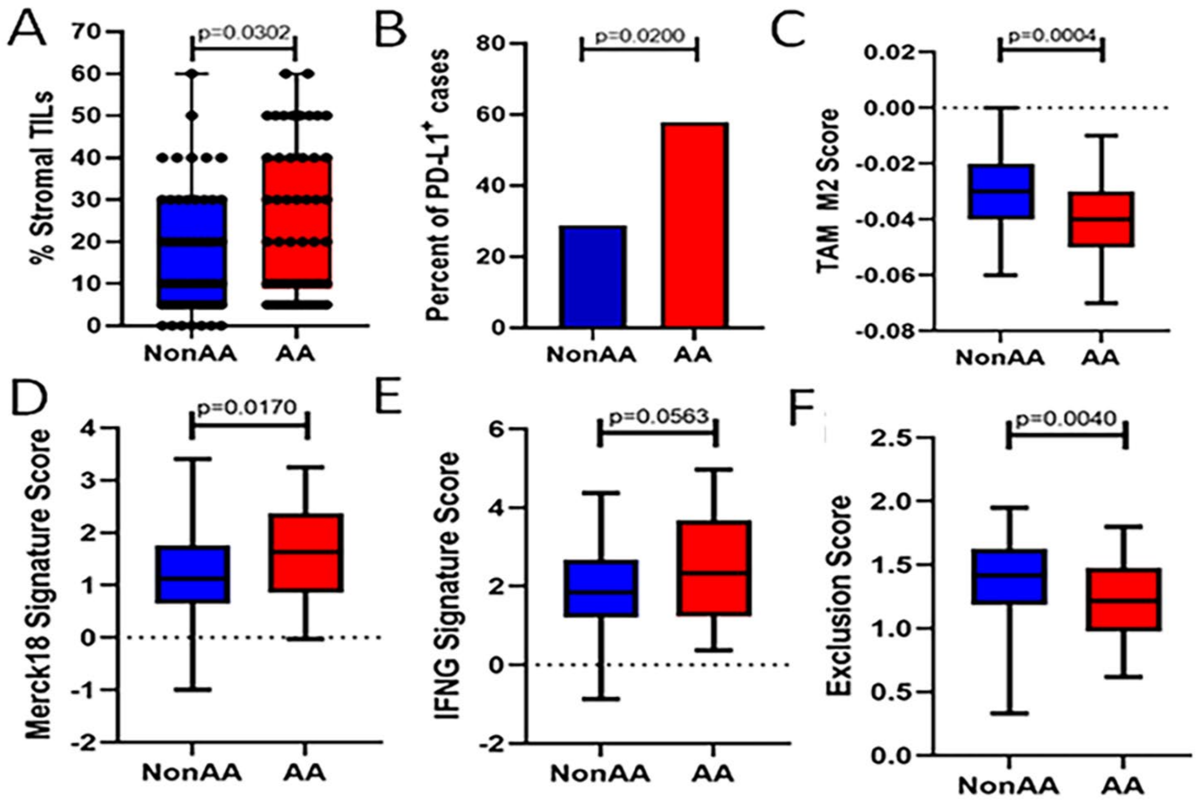

Supplementary Figure 7. **Significantly different immune variables in TNBC in Non-African American (NonAA) and African American (AA) patients including stromal tumor infiltrating lymphocytes (sTILs), PD-L1 immunohistochemistry, and immune gene expression scores from Tumor Immune Dysfunction and Exclusion (TIDE) analysis.** (A) TIL percent. (B) PD-L1 positivity rates by SP142 immunohistochemistry. (C) Tumor-associated macrophage M2 (TAM M2), (D) Immune inflamed (Merck18), (E) interferon gamma (IFNG), and (F) T cell Exclusion scores. Nominal p-values unadjusted for multiple comparisons are shown. P-values from Mann-Whitney Test.

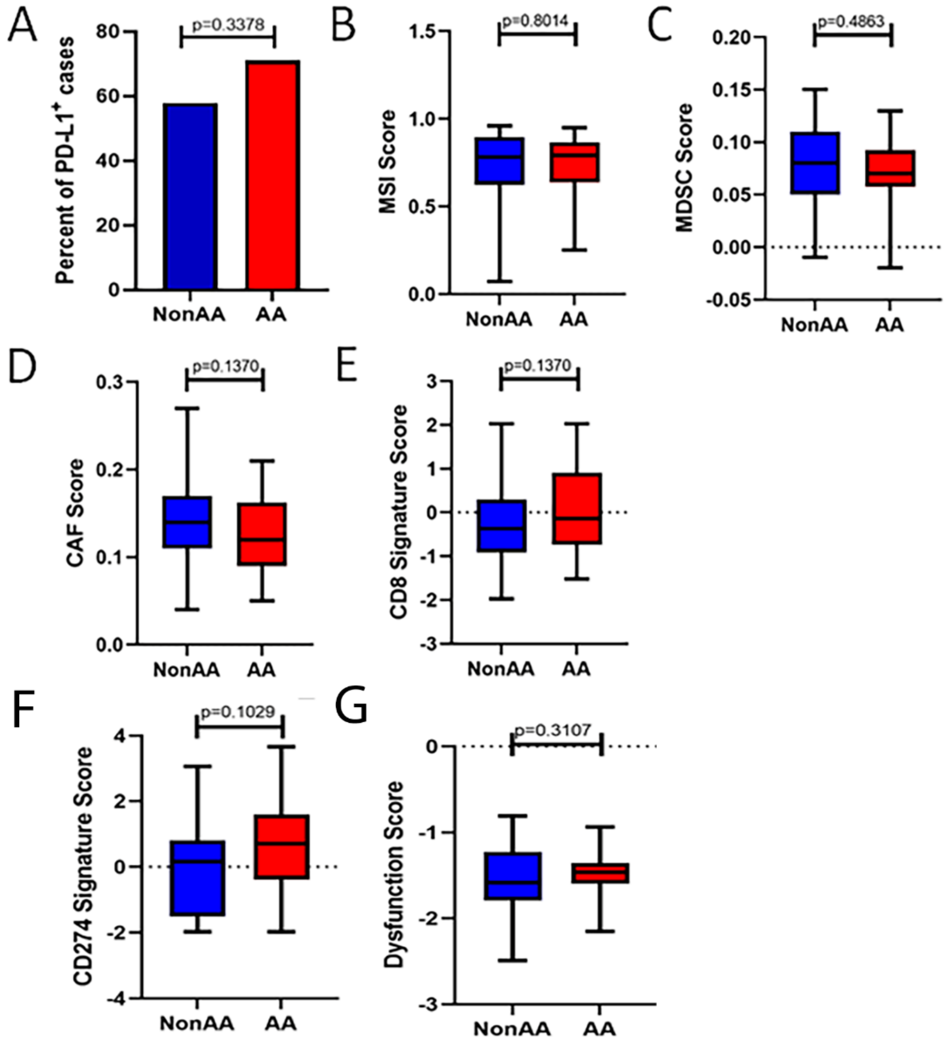

Supplementary Figure 8. **Immune parameters not significantly different between Non-African American (NonAA) and African American (AA) TNBC patients.** (A) PD-L1 SP263 immunohistochemistry analyzed as a contingency table using the Fisher's Exact Test. Immune scores from Tumor Immune Dysfunction and Exclusion (TIDE) analysis: (B) Microsatellite instability (MSI), (C) Myeloid-derived suppressor cells (MDSC), (D) Cancer-associated fibroblasts (CAF), (E) CD8 expression, (F) PD-L1 (CD274) expression scores, and (G) Immune Dysfunction. P-values from Mann-Whitney Test.

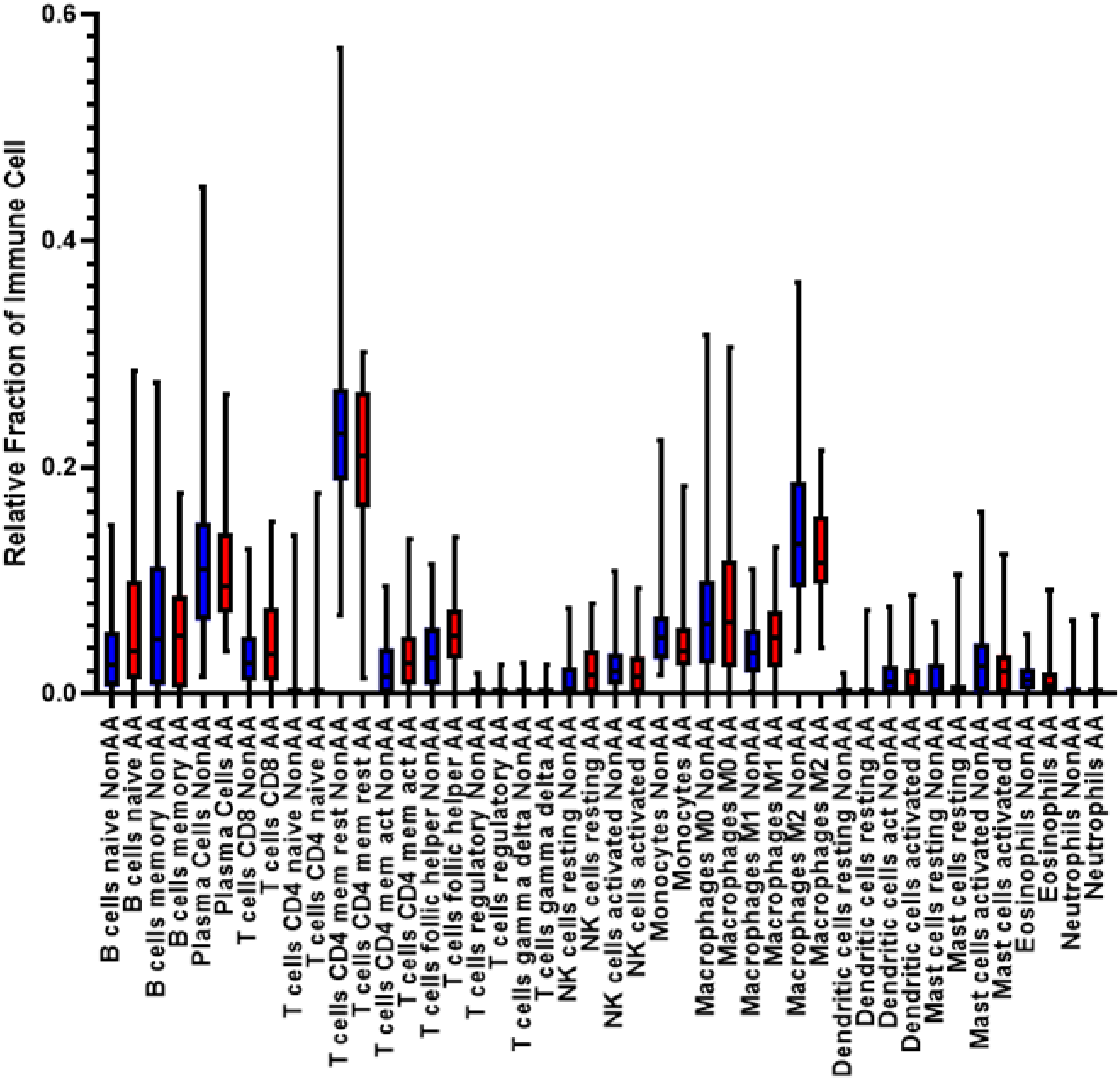

Supplementary Figure 9. **CIBERSORTx cell type abundancies in TNBC in Non-African American (NonAA) and African American (AA) patients**. Blue = NonAA. Red = AA.

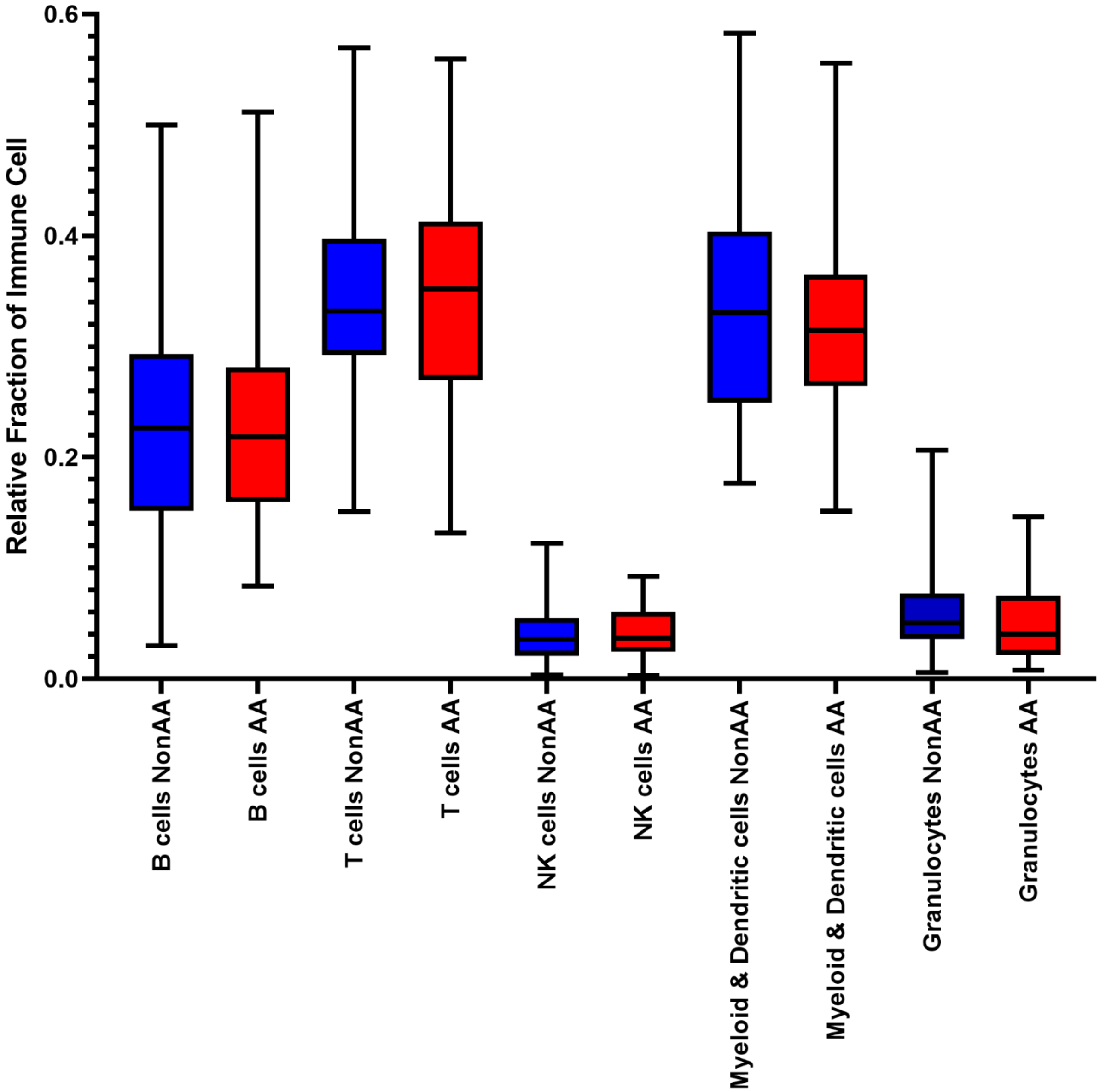

Supplementary Figure 10. CIBERSORTx Groups in TNBC Non-African American (NonAA) and African American (AA) patients. Blue = NonAA. Red = AA. P-values from Kruskal–Wallis Test.

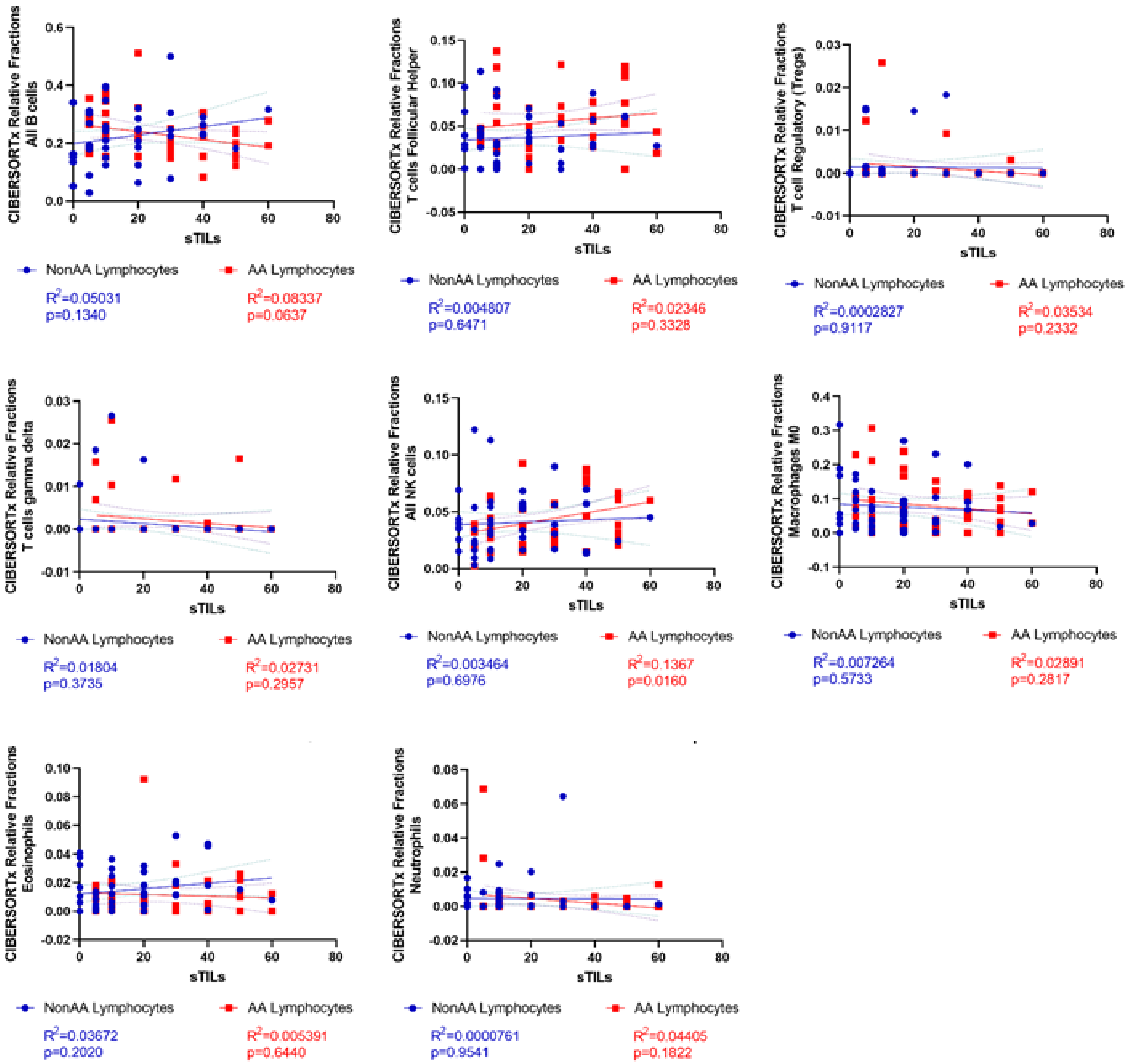

Supplementary Figure 11. Correlations between histological stromal TILs and deconvoluted CIBERSORTx immune cell fractions in TNBC in Non-African American (NonAA) and African American (AA) patients. Blue = NonAA. Red = AA. P-values from Linear Regression.

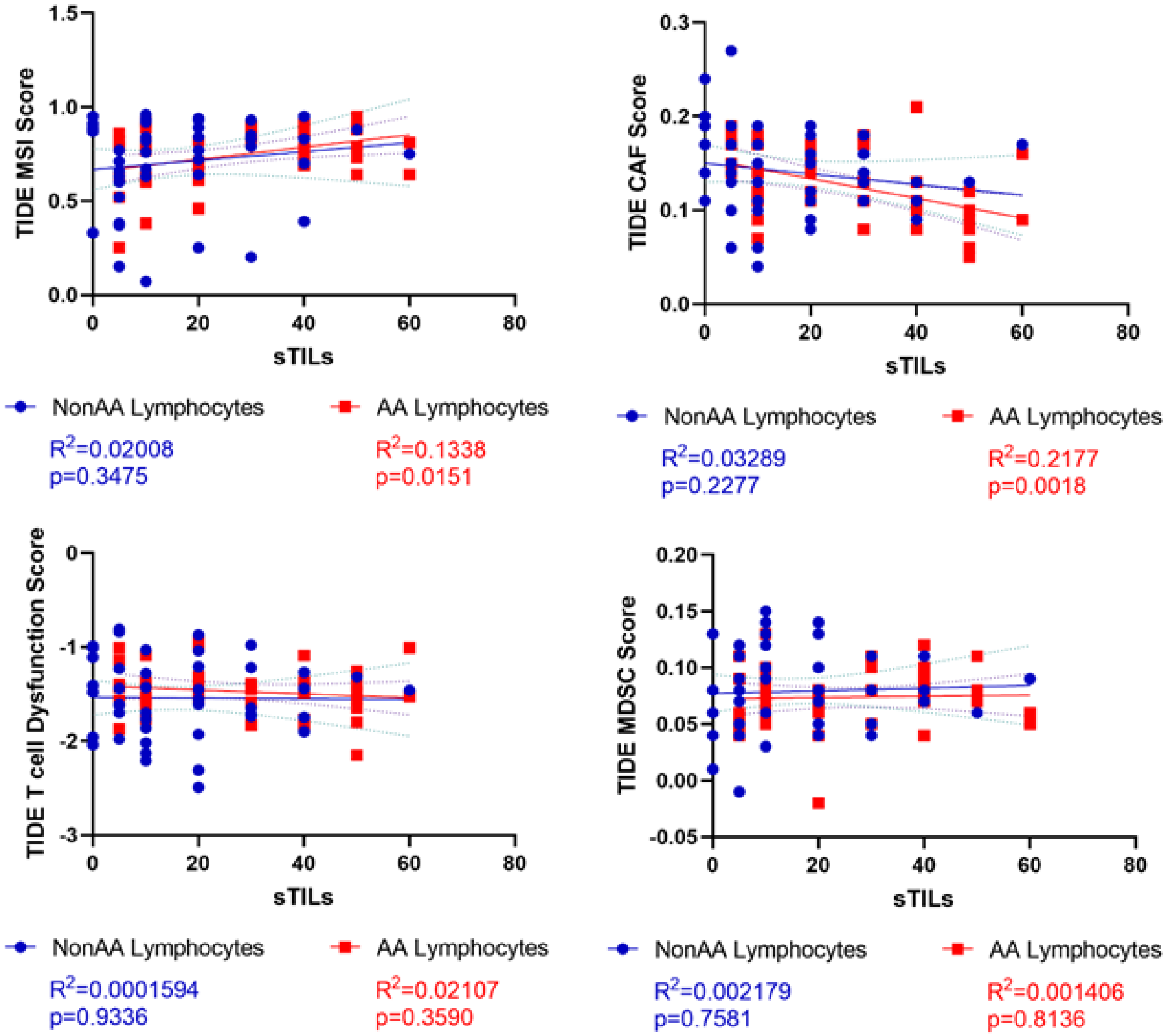

Supplementary Figure 12. Correlations between histological stromal TILs and TIDE signature scores in TNBC in Non-African American (NonAA) and African American (AA) patients. Blue = NonAA. Red = AA. P-values from Linear Regression.

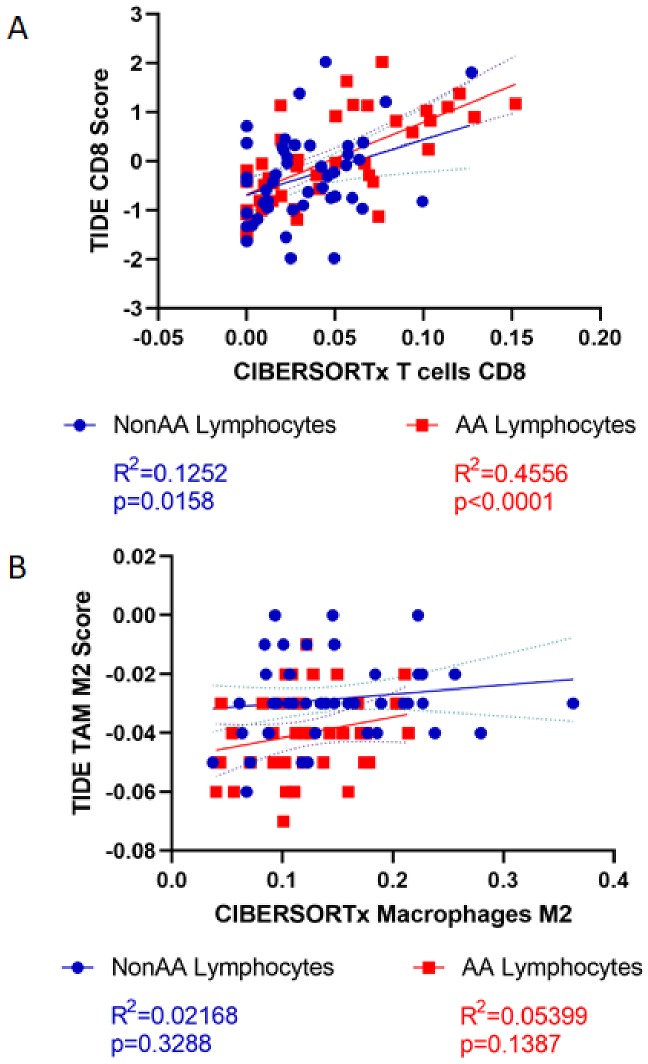

Supplementary Figure 13. Correlations between similar populations deconvoluted with CIBERSORTx or TIDE signature scores in TNBC in Non-African American (NonAA) and African American (AA) patients. (A) CD8. (B) M2 Macrophages. Blue = NonAA. Red = AA. P-values from Linear Regression.

References

1 Park, J. H. *et al.* Prognostic value of tumor-infiltrating lymphocytes in patients with early-stage triple-negative breast cancers (TNBC) who did not receive adjuvant chemotherapy. *Annals of oncology : official journal of the European Society for Medical Oncology / ESMO* **30**, 1941-1949, doi:10.1093/annonc/mdz395 (2019).
